# Deep Transcriptome Sequencing of Pediatric Acute Myeloid Leukemia Patients at Diagnosis, Remission and Relapse: Experience in 3 Malaysian Children in a Single Center Study

**DOI:** 10.3389/fgene.2020.00066

**Published:** 2020-02-27

**Authors:** Siti Hawa Osman, Nadiah Abu, Habsah Aziz, Yock Ping Chow, Wan Fahmi Wan Mohamad Nazarie, Nurul-Syakima Ab Mutalib, Hamidah Alias, Rahman Jamal

**Affiliations:** ^1^ UKM Medical Molecular Biology Institute, The National University of Malaysia, Cheras, Malaysia; ^2^ Department of Pediatrics, UKM Medical Centre, Faculty of Medicine, The National University of Malaysia, Cheras, Malaysia

**Keywords:** acute myeloid leukemia, pediatric, RNA-Seq, relapse, chemotherapy

## Introduction

Among the many types of leukemia, acute myeloid leukemia (AML) affects 20% of diagnosed hematological malignancies in pediatric patients (Meshinchi and Arceci, 2007; de Rooij et al., 2015). Standard chemotherapy regimen remains as the first line treatment for pediatric AML, however nearly 40% of AML patients may suffer from relapse and eventually die from the disease (de Rooij et al., 2015). Similarly, it has been reported that 50% of the pediatric AML relapsed within 12–18 months of diagnosis and 45% of those relapsed were not expected to survive (Creutzig et al., 2014). Despite advances in cytogenetic analysis through fluorescence *in situ* hybridization and multiplex PCR, there is still a need for a better and comprehensive molecular profiling. For instance, microarray has long been used to study the gene expression profiles of AML patients. The different profile of gene expression has enabled clinicians to tailor better treatment for patients and predict whether patients have the tendency to relapse (Goswami et al., 2009). In a recent study, Handschuch et al. reported that three genes, ANXA3, S100A9, and WT1 can differentiate between different prognostic types of AML (Handschuh et al., 2018). The study outcome was in agreement with another study conducted by Shimada et al. (2012), where a high expression of WT1 gene showed prognostic impact in pediatric AML (Shimada et al., 2012). Another study by Jo et al. (2015) reported that high expression of EVI1 and MEL1 could predict the prognosis of pediatric AML (Jo et al., 2015). However, none of the biomarkers identified from these studies have been translated into clinical use. Therefore, the search continues for additional promising biomarkers, notably novel transcripts, novel fusion genes and non-coding RNAs which are not represented in the microarray platform. Transcriptome sequencing through next generation sequencing represents an effective approach to discover new genetic information on gene expression which may contribute to tumorigenesis. Notably, several novel and rare fusion transcripts have been identified from AML patients *via* RNA-sequencing (Padella et al., 2015). A recent study combining whole genome sequencing, whole exome sequencing and RNA sequencing in pediatric cancers has identified 240 pathogenic variants with increased sensitivity (Rusch et al., 2018). Previous studies in relapsed AML have shown that the cells acquired additional genetic mutations that were either different or evolved from subclones of diagnostic blasts cells (Padella et al., 2015; Rusch et al., 2018). Nevertheless, little is known about the genetic changes at the transcriptomic level at diagnostic, remission and relapse stages of the same patients, especially in the Malaysian population.

## Value of Data

- AML is the second commonest hematological malignancy affecting children worldwide and more research at transcriptome level is needed to help to improve the survival rates.
- This data is of value to understand further the molecular landscape underpinning *de novo* and relapsed pediatric AML in the Malaysian population.
- Most of the data available only report the molecular profiles at diagnosis and relapse stages. In this study we performed the sequence at remission as well. This finding is important for researchers to understand the changes in the progression of the disease at all stages.
- Deep transcriptome sequencing will allow users to not only obtain the gene expression profile, but also for fusion gene identification and mutation analysis.

## Data

In this experimental design, we successfully sequenced the RNA of three Malaysian pediatric AML patients at three different stages; diagnosis, remission, and relapse. **Table 1** displays the demographic information of the patients involved in this study. Two out of three patients had a RUNX1-RUNXITI translocation while the other patient was cytogenetically normal. The RUNXI-RUNXITI translocation is one of the most widely identified chromosomal aberration in AML patients. Moreover, it has been reported that patients with this translocation have a higher change of gaining relapse (Christen et al., 2019). All patients relapsed within one year after remission. Based on our next generation sequencing results, for PAML1, the relapse (RL) sample yielded the highest number of reads but with lower total percentage of mapped reads at 78.47% as compared to diagnosis (DX) and remission (RM) stages with 81.33% and 80.72%, respectively. For PAML2 and PAML3, the remission samples resulted in the highest number of reads and percentage total mapped as compared to diagnosis and relapse samples. Collectively, as shown in **Table 2**, PAML3 had relatively higher reads as compared to PAML 1 and PAML2 for all three stages. This subsequently resulted in a higher percentage of total mapped reads in PAML3. The raw sequences for each sample were submitted to Sequence Read Archive (SRA), with accession number PRJNA509497. All the samples were at least mapped >73% to the exonic region of the genome except for PAML1-DX sample.

**Table 1:** Demographic data of the pediatric patients involved in this study.

Patient	Gender	Age at diagnosis	Ethnicity	Cytogenetic profile	Duration to relapse (Months)	Chemotherapy regimen
PAML1	Female	8	Indian	Normal	9	FLAG
PAML2	Female	16	Malay	t(8;21)(q22;q22) RUNX1-RUNX1T1 Translocation	11	FLAG
PAML3	Male	17	Malay	t(8;21)(q22;q22) RUNX1-RUNX1T1 Translocation	7	FLAG

**Table 2:** Quality assessment of reads obtained from each PAML patient.

	PAML1			PAML2			PAML3		
	DX	RM	RL	DX	RM	RL	DX	RM	RL
Raw reads	247,961,018	234,838,958	276,018,254	244,219,878	264,979,028	235,353,546	316,984,346	351,914,556	321,428,064
Clean reads	239,742,412	226,525,696	267,790,788	157,787,792	180,615,972	179,874,102	307,646,810	341,058,194	312,010,790
Total mapped	194,973,920 (81.33%)	182,859,720 (80.72%)	210,128,031 (78.47%)	138,318,334 (87.66%)	162,003,152 (89.69%)	160,435,318 (89.19%)	289,204,380 (94.01%)	324,845,708 (95.25%)	294,019,765 (94.23%)

**Figure 1:**
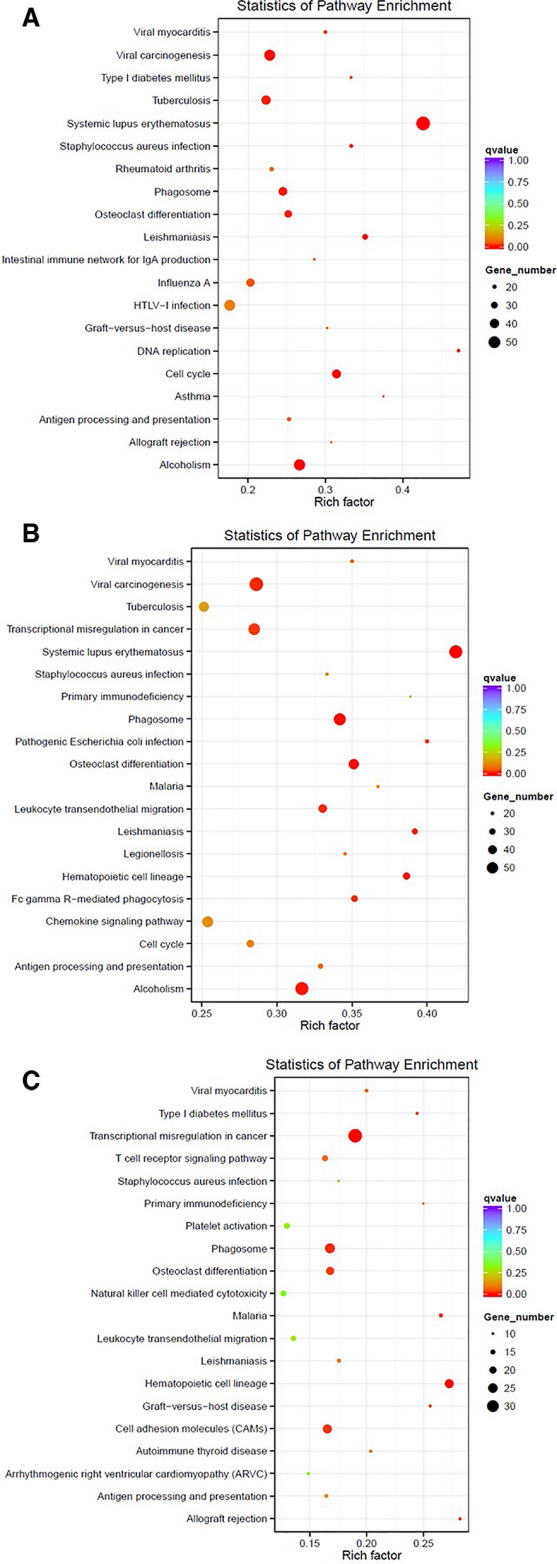
Enriched pathways based on the KEGG database for PAML1 using different comparisons: **(A)** Relapse vs. Diagnosis; **(B)** Diagnosis vs. Remission; and **(C)** Relapse vs. Remission.

One of the analyses that can be used with this data is the identification of differentially expressed genes. For instance, here, we compare the gene expression profile for PAML1, using three different group comparisons (1) Diagnosis vs. Relapse, (2) Diagnosis vs. Remission, and (3) Relapse vs, Remission (Further analysis of PAML2 and PAML3 can be found in **Data Sheet 1**). **Table 3** lists the top 10 differentially expressed genes in all comparison groups. The top genes were different between each group which shows that the regulation of gene expression in each stage of the patient is also distinct. Furthermore, we performed KEGG pathway enrichment analysis based on the differentially expressed genes, as shown in **Figure 1**. In the relapse vs. diagnosis comparison, the most enriched pathway is the systemic lupus erythematosus pathway followed by viral carcinogenesis, and cell cycle. Similarly, the same pathways were also enriched in the diagnosis vs. remission comparison. There have been studies reporting on the association between systemic lupus erythematosus, or any autoimmune diseases with the risk of developing AML (Tsunematsu et al., 1984; Ramadan et al., 2012). Nevertheless, AML is usually attributed as the effect of administering cytotoxic drugs in autoimmune diseases (Ramadan et al., 2012). The causal link between AML and systemic lupus erythematosus needs further elucidation, even though, there is indeed a link in terms of molecular structure between these two diseases. Moreover, the cell cycle pathway has also been previously reported to be involved in the hematopoietic landscape of AML (Yagi et al., 2003; Handschuh, 2019). Whereas for the relapse vs remission comparison, the most enriched pathway is the transcriptional misregulation in cancer. This was in concordance with a different study conducted on AML, where the authors found that this pathway was among the topmost enriched pathways (Roushangar and Mias, 2019). This observation also implicates that the genes that are being regulated from remission to relapse are different than at the diagnosis stage. Nevertheless, these data need to be used with caution since it is a high throughput sequencing, further validation is recommended.

**Table 3:** Top 10 differentially expressed genes in PAML1 for three different comparison groups.

Comparison	Gene	Log_2_ Fold Change	p-value (< 0.05)
Relapse vs. Diagnosis	UPK3A	9.4852	5.69E-05
	DHRS9	8.5592	2.82E-19
	CES1	7.7764	1.18E-06
	MS4A6A	7.7741	2.16E-167
	MPEG1	7.6023	1.52E-267
	CPED1	7.5904	3.39E-10
	MS4A1	7.5313	3.97E-12
	ARHGEF10L	7.4565	1.23E-11
	CD1D	7.3666	1.24E-18
	LGALS2	7.3432	6.49E-15
Relapse vs. Remission	SAGE1	6.3811	6.66E-10
	PRAME	6.2109	3.64E-05
	CLEC2A	6.2008	2.53E-14
	PCDH7	6.1402	1.49E-57
	GXYLT2	6.0147	1.77E-61
	SKIDA1	5.8422	2.28E-34
	TFPI2	5.7403	2.52E-09
	NDN	5.5787	1.91E-05
	MRC1L1	5.4777	1.28E-12
	ANKRD18B	5.4641	1.63E-27
Diagnosis vs. Remission	PI15	8.1707	2.82E-09
	TTLL6	7.8833	3.85E-06
	GPR32	7.7459	1.52E-07
	SOX1	7.6051	2.83E-22
	MDGA1	7.1817	0.000177
	MUC19	7.0129	1.40E-11
	SLC52A3	6.9089	4.47E-05
	C11orf87	6.8654	0.000192
	COL4A5	6.7879	9.12E-28
	GALNT18	6.6336	0.000179

## Material and Methods

### Pediatric AML Sample Collection and Mononuclear Cells Isolation

Three sets of diagnostic, remission and relapse samples (n = 3) derived from the bone marrow of 3 patients were obtained. This study was reviewed and approved by the Medical Research Ethics Committee of Universiti Kebangsaan Malaysia. Written informed consent was obtained from all parents. The percentage of blasts at diagnosis and relapse were >50%, while at remission the blasts percentage were <5%. PAML1 had a cytogenetically normal karyotype, while PAML2 and PAML3 had t(8,21) translocations. Mononuclear cells (MNC) were isolated using the Ficoll-Paque (Invitrogen, USA) method.

### RNA Extraction

Total RNA from isolated MNC was extracted and purified according to the standard protocol of AllPrep DNA/RNA/miRNA Universal Kit (Qiagen, Germany). The quality and quantity of the total RNA were checked using NanoDrop ND-1000 (NanoDrop Technologies, USA) and Qubit 2.0 Fluorometer (Invitrogen, Life Technologies, USA). Total RNA integrity was then assessed using the Agilent 2100 Bio-Analyzer (Agilent Technologies, USA). Only those bone marrow aspirate (BMA) with RNA integrity number (RIN) > 7.0 were included in transcriptome sequencing (RNA-Seq).

### Library Preparation and RNA Sequencing

One microgram of total RNA was used to remove ribosomal RNA (rRNA) using the Ribo-Zero rRNA Removal Kit (Illumina, USA). Purified rRNA-depleted RNA was subjected to RNA library preparation using the ScriptSeqTM v2 RNA-Seq Library Preparation Kit according to the manufacturer instructions. After library construction, the library was diluted to 1.5 ng/µl after preliminary quantitation by Qubit 2.0 and insert size by Agilent 2100. Qpcr was used to accurately quantify the library effective concentration (> 2nM), in order to ensure the library quality. The libraries were then sequenced on Illumina HiSeq 2500 (Illumina, USA).

### Bioinformatics Analysis

Paired-end sequences were individually obtained as FASTQ files from the images by CASAVA base recognition software. We later filtered the raw reads to remove adaptor sequences, reads containing undetermined bases >10%, low quality reads having QScore of over 50% bases of the read is < = 5. After obtaining the clean reads, we further mapped the reads against the human reference genome (GRCh38) using TopHat2 (Kim et al., 2013). For the differentially expressed genes analysis, the read count was adjusted using trimmed mean of M values (TMM), then the differential expression analysis was conducted using the DEGseq R package (Wang et al., 2010). The pathway enrichment analysis was performed based on the KEGG database (Kanehisa et al., 2019). KEGG enriched pathway results were demonstrated by using Scatterplot. The KEGG enriched pathway results were evaluated by the Rich factor value, Qvalue and the number of differentially expressed genes involved in the enriched pathways. The rich factor value refers to the ratio of the number of differentially expressed genes in the pathway and the number of all genes annotated in the same pathway. The bigger the value of the rich factor is, the more significant the enrichment degree is.

## Data Availability Statement

All of the sequencing reads from this project have been uploaded to NCBI with the BioProject ID PRJNA509497 (https://www.ncbi.nlm.nih.gov/sra/?term=PRJNA509497).

## Ethics Statement

The studies involving human participants were reviewed and approved by National University of Malaysia Ethics Committee. Written informed consent to participate in this study was provided by the participants' legal guardian/next of kin.

## Author Contributions

NA, WN, SO drafted the manuscript. SO, HAz, YC, and HAl collected the samples and performed the experimental work. NA, WN, and N-SM performed the data analysis. HAl and RJ provided critical feedback and input.

## Funding

This project was supported by the Fundamental Research Grant Scheme (FRGS) provided by the Ministry of Higher Education Malaysia, with the grant ID FRGS/1/2015/SKK08/UKM/03/2.

## Conflict of Interest

The authors declare that the research was conducted in the absence of any commercial or financial relationships that could be construed as a potential conflict of interest.
