# Supplementary material for: Deep Transcriptome Sequencing of Pediatric Acute Myeloid Leukemia Patients at Diagnosis, Remission and Relapse: Experience in 3 Malaysian Children in a Single Center Study
Source: Front Genet. 2020 Feb 27;11:66. doi: 10.3389/fgene.2020.00066 (PMC7056821; doi:10.3389/fgene.2020.00066)
Supplement: Supplementary file 1 [file DataSheet_1.docx]

**Supplementary Information**

**Table S1: Top 10 differentially expressed genes for each comparison group for PAML2**

| Comparison | Gene | Log_2_ Fold Change | p-value (< 0.05) |
| --- | --- | --- | --- |
| Relapse vs Diagnosis | GPR32 | 7.855 | 5.74E-05 |
|  | CFI | 6.662 | 2.66E-26 |
|  | PLEKHG4B | 6.431 | 1.79E-05 |
|  | THSD7A | 6.14 | 4.61E-17 |
|  | MCAM | 5.7452 | 2.44E-18 |
|  | NEGR1 | 5.2908 | 6.82E-20 |
|  | APP | 5.1756 | 3.26E-47 |
|  | VCAM1 | 5.0339 | 1.61E-05 |
|  | BEND4 | 4.9795 | 5.79E-09 |
|  | ATP9A | 4.9744 | 6.45E-05 |
| Relapse vs Remission | ARC | 8.7052 | 7.61E-05 |
|  | BAI1 | 8.3294 | 0.000389 |
|  | BCL2L14 | 7.7346 | 2.62E-07 |
|  | ADGRL3 | 7.728 | 4.47E-16 |
|  | DLK1 | 7.6817 | 7.71E-13 |
|  | MAPK11 | 7.6411 | 0.000401 |
|  | GPR32 | 7.3187 | 3.48E-05 |
|  | PNMA5 | 7.2921 | 2.78E-39 |
|  | PCDHGB2 | 7.2537 | 1.58E-06 |
|  | VLDLR | 7.0682 | 8.57E-41 |
| Diagnosis vs Remission | NTRK1 | 8.2276 | 1.90E-07 |
|  | MSLN | 7.734 | 3.47E-20 |
|  | ENPP3 | 7.6522 | 6.66E-16 |
|  | PNMA5 | 7.3339 | 7.67E-37 |
|  | POU4F1 | 7.2991 | 4.38E-120 |
|  | PRAME | 7.2755 | 1.29E-84 |
|  | DNAH8 | 7.2491 | 2.18E-49 |
|  | CD19 | 7.1655 | 0.000166 |
|  | PCDHGB2 | 6.9743 | 2.74E-05 |
|  | TCN1 | 6.9533 | 3.58E-162 |

**Table S2: Top 10 differentially expressed genes for each comparison group for PAML3**

| Comparison | Gene | Log_2_ Fold Change | p-value (< 0.05) |
| --- | --- | --- | --- |
| Relapse vs Diagnosis | COL4A5 | 1.01E-13 | 8.9549 |
|  | LOXL1 | 6.38E-24 | 8.1985 |
|  | DLK1 | 2.93E-31 | 7.779 |
|  | FGFR2 | 1.61E-05 | 7.759 |
|  | SCN2A | 1.59E-08 | 7.75 |
|  | SLITRK6 | 0.000151 | 7.3456 |
|  | DSC2 | 4.17E-14 | 7.233 |
|  | GLI2 | 3.11E-08 | 7.0165 |
|  | STOX2 | 5.41E-14 | 6.7446 |
|  | RPS17 | 3.49E-29 | 6.5776 |
| Relapse vs Remission | RPS17 | 6.5823 | 5.39E-29 |
|  | DLK1 | 3.7861 | 5.81E-27 |
|  | C2orf54 | 3.7312 | 3.87E-06 |
|  | MFAP4 | 3.3983 | 5.15E-15 |
|  | CCDC8 | 3.3147 | 9.21E-05 |
|  | ZDHHC11 | 3.2574 | 0.000294 |
|  | MFAP2 | 3.1508 | 9.07E-05 |
|  | HPGDS | 3.1424 | 1.51E-12 |
|  | GTSF1 | 3.06 | 6.87E-14 |
|  | FIBCD1 | 3.0198 | 7.42E-11 |
| Diagnosis vs Remission | TCN1 | 7.2018 | 3.41E-14 |
|  | NR5A2 | 7.0933 | 0.000121 |
|  | CCNA1 | 5.9893 | 1.11E-21 |
|  | MAMDC2 | 5.9281 | 1.63E-14 |
|  | HR | 5.8685 | 1.27E-06 |
|  | MAPK15 | 5.828 | 4.18E-09 |
|  | TPPP3 | 5.7362 | 1.78E-18 |
|  | MSLNL | 5.6658 | 0.000226 |
|  | ST18 | 5.5685 | 1.01E-12 |
|  | WT1 | 5.4889 | 0.000323 |


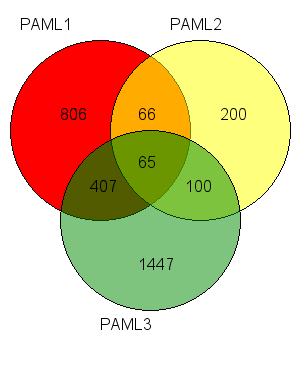


**Figure S1: Overlapping differentially expressed genes for each of the PAML patients for the Relapse vs Diagnosis comparison**

**Table S3: Expression of key genes related to AML in all three patients at all stages**

| Gene | PAML1 | | | PAML2 | | | PAML3 | | |
| --- | --- | --- | --- | --- | --- | --- | --- | --- | --- |
|  | Relapse vs Diagnosis | Relapse vs Remission | Diagnosis vs Remission | Relapse vs Diagnosis | Relapse vs Remission | Diagnosis vs Remission | Relapse vs Diagnosis | Relapse vs Remission | Diagnosis vs Remission |
| ANXA3 | n.s | -2.8711 | n.s | n.s | -2.8505 | n.s | n.s | n.s | n.s |
| S100A9 | 6.859 | -1.4029 | -8.1786 | -2.7337 | -4.6115 | -1.9124 | -3.9284 | -8.0464 | -4.0397 |
| WT1 | -3.4939 | n.s | 7.2812 | n.s | n.s | n.s | n.s | n.s | 5.4889 |
| EV11 | n.s | n.s | n.s | n.s | n.s | n.s | n.s | n.s | n.s |
| MEL1 | n.s | n.s | n.s | n.s | n.s | n.s | n.s | n.s | n.s |
